# Guillain-Barré Syndrome Following Seasonal Coronavirus (HCoV-229E) Infection: A Case Managed Without Intensive Care

**DOI:** 10.7759/cureus.107360

**Published:** 2026-04-19

**Authors:** Goitom Weldearegay, Tigran Aghabekyan, Ali Arif, Jerome Salvani

**Affiliations:** 1 Internal Medicine, SUNY Downstate University Hospital, New York, USA

**Keywords:** coronavirus‑associated neurologic complications, guillain-barré syndrome, human coronavirus 229e, neuromuscular weakness, peripheral nerve demyelination

## Abstract

Guillain-Barré syndrome (GBS) is an acute immune-mediated polyradiculoneuropathy often preceded by infection. We report the case of a 49-year-old man presenting with subacute, progressive, symmetric ascending weakness involving the lower and distal upper extremities, ultimately diagnosed with GBS, triggered by human coronavirus 229E infection. The patient was managed with intravenous immunoglobulin (IVIG) and close respiratory monitoring in a stepdown unit without progression to respiratory failure or need for intensive care unit (ICU) admission. This case highlights diagnostic considerations, the role of electrophysiologic testing, and ICU triage decision-making in suspected GBS.

## Introduction

Guillain-Barré syndrome (GBS) most commonly presents with rapidly progressive, symmetric weakness accompanied by hyporeflexia or areflexia, with variable sensory and autonomic involvement. Antecedent infections are well‑established triggers. Although GBS associated with SARS‑CoV‑2 has been increasingly reported, less attention has been directed toward non‑SARS seasonal coronaviruses. Close monitoring for respiratory compromise is essential, as up to 30% of patients require ventilatory support [1]. Consequently, early recognition and vigilant observation for respiratory decline are critical. This case highlights the development of GBS following human coronavirus 229E (HCoV‑229E) infection and illustrates management guided by clinical and physiologic assessment without the need for ICU admission.

## Case presentation

A 49‑year‑old male with a history of untreated hypertension presented with progressive bilateral lower‑extremity weakness. He had been in his usual state of health until January 2026, when he developed heaviness and weakness in both feet. Symptoms progressed proximally over 48 hours, leading to difficulty ambulating. The following day, he noted distal upper‑extremity involvement affecting both hands, impairing fine motor tasks and driving.

He denied pain, paresthesias, sensory loss, bowel or bladder dysfunction, fever, chills, recent gastrointestinal illness, or respiratory symptoms. He reported receiving influenza and COVID‑19 vaccinations approximately three months prior. He initially presented to an outside emergency department and was discharged after basic laboratory evaluation. Continued progression prompted outpatient neurology evaluation, where electromyography (EMG) demonstrated absent bilateral peroneal and tibial motor responses with acute denervation in the lower extremities and paraspinal muscles. He was referred to University Hospital of Brooklyn for further evaluation of suspected GBS.

On admission, he was afebrile and hemodynamically stable, blood pressure was initially 176/101 mmHg and improved to 149/92 mmHg without intervention. His heart rate was 89 bpm, respiratory rate 18 breaths per minute, and oxygen saturation 98% on room air. He was alert and oriented without distress. Cardiopulmonary examination was unremarkable. Neurologic examination showed intact mental status and cranial nerves. Motor testing revealed distal‑predominant weakness in the upper extremities (shoulders 4+/5, wrists 4/5, fingers 3/5) and severe distal weakness in the lower extremities (ankle dorsiflexion and plantarflexion 0/5) with relatively preserved proximal strength (hips and knees 4+/5). Reflexes were diffusely diminished to absent, including absent ankle reflexes and reduced patellar responses. Sensation to light touch, pinprick, and temperature was intact. Coordination was preserved, though he was unable to ambulate independently.

**Figure 1:**
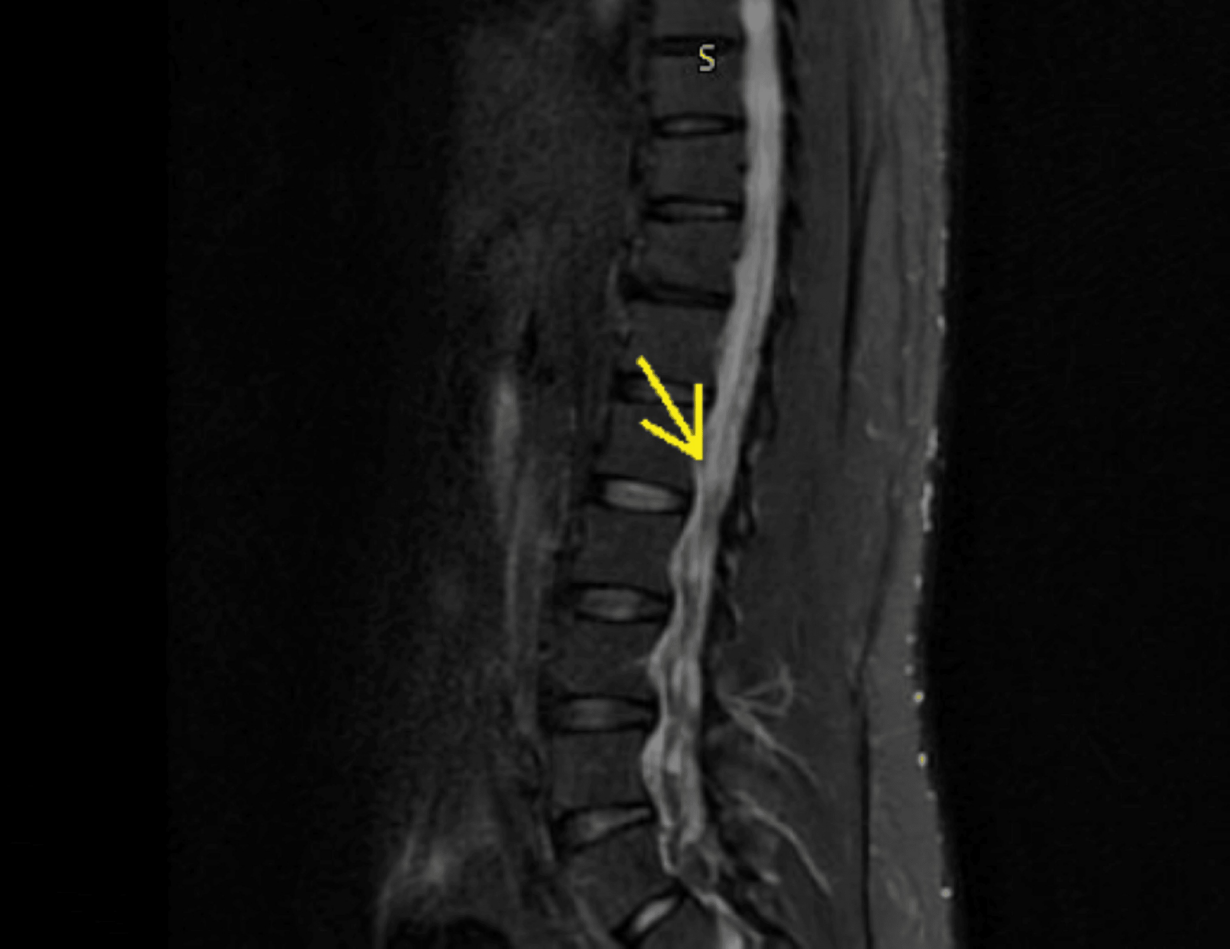
Lumbar spine MRI demonstrating normal alignment without evidence of neural compression, bone fracture, or cerebrospinal fluid (CSF) leak (yellow arrow) S in the picture represents sagittal plane.

**Figure 2:**
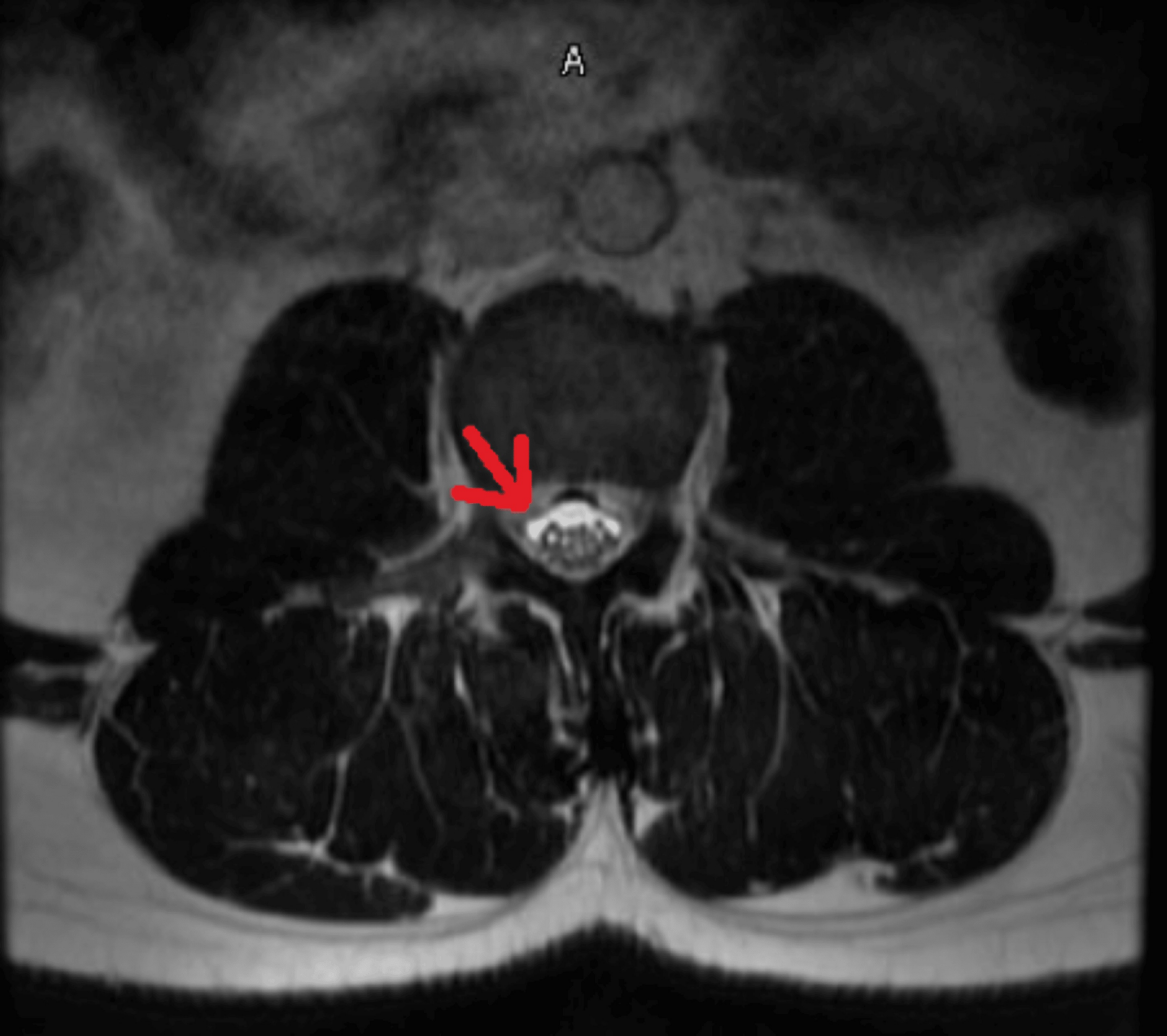
Spine MRI (T1 weighted post contrast with fat suppression) showing symmetrical bilateral nerve root enhancement (arrow). A in the figure represents axial plane.

Laboratory evaluation showed mild hyperkalemia (peak 5.8 mmol/L) without electrocardiographic changes, which resolved with treatment. Complete blood count and renal function were within normal limits. Creatine kinase was mildly elevated (peak 327 U/L), and inflammatory markers were increased (ESR 55 mm/hr, CRP 5.7 mg/L) (Table 1). Cerebrospinal fluid (CSF) analysis demonstrated albuminocytologic dissociation, with mildly elevated protein (44 mg/dL), 0 WBCs, normal glucose, and negative Gram stain and cultures (Table 2). Given the electrodiagnostic findings from the outside facility, there was concern for an acute motor axonal neuropathy variant of Guillain-Barré syndrome. MRI of the cervical, thoracic, and lumbar spine with contrast showed lumbar nerve root enhancement without compressive or structural pathology (Figures 1, 2). Respiratory viral molecular testing was positive for human coronavirus 229E, while SARS‑CoV‑2 and other respiratory pathogens were not detected (Table 3). Serial respiratory assessments demonstrated preserved pulmonary mechanics, with negative inspiratory force consistently greater than -40 cm H₂O and vital capacity approximately 3.27 L.

**Table 1:** Admission Laboratory Studies (Complete Blood Count and Comprehensive Metabolic Panel) BUN - Blood Urea Nitrogen, AST - Aspartate Aminotransferase, ALT - Alanine Aminotransferase, ESR - Erythrocyte Sedimentation Rate, CRP - C‑Reactive Protein, WBC - White Blood Cell count

Test	Result	Reference Range
Sodium	140 mmol/L	135-145
Potassium	5.8 mmol/L (resolved to 4.4)	3.5-5.0
Chloride	107 mmol/L	98-107
CO₂ (Bicarbonate)	24 mmol/L	22-29
BUN	11.1 mg/dL	7-20
Creatinine	0.70 mg/dL	0.6-1.3
Glucose	99 mg/dL	70-110
AST	50 U/L	10-40
ALT	38 U/L	7-56
Alkaline Phosphatase	51 U/L	44-147
Total Bilirubin	0.7 mg/dL	0.1-1.2
Albumin	5.3 g/dL	3.5-5.0
Creatine Kinase	327 U/L	30-200
ESR	55 mm/hr	<20
CRP	5.7 mg/L	<3
WBC	7.13 ×10³/µL	4.0-11.0
Hemoglobin	15.8 g/dL	13.5-17.5
Platelets	257 ×10³/µL	150-400

**Table 2:** Cerebrospinal Fluid (CSF) Analysis RBC - Red Blood Cells, WBC - White Blood Cells

Parameter	Result	Reference Range
Appearance	Clear, colorless	Clear
WBC	0 cells/µL	0-5
RBC	0 cells/µL	0
Protein	44 mg/dL	15-40
Glucose	66 mg/dL	40-70
Gram Stain	No organisms detected	-
Culture	No growth	-

**Table 3:** Respiratory Viral Panel (Molecular Testing)

Pathogen	Result
Coronavirus 229E	Detected
Coronavirus HKU1	Not detected
Coronavirus NL63	Not detected
Coronavirus OC43	Not detected
SARS-CoV-2 RNA	Not detected
Influenza A	Not detected
Influenza B	Not detected
Respiratory Syncytial Virus	Not detected
Parainfluenza 1	Not detected
Parainfluenza 2	Not detected
Parainfluenza 3	Not detected
Parainfluenza 4	Not detected
Adenovirus	Not detected
Human Metapneumovirus	Not detected
Bordetella pertussis	Not detected
Mycoplasma pneumoniae	Not detected
Chlamydia pneumoniae	Not detected

Given the classic clinical presentation, electrophysiologic findings, and supportive cerebrospinal fluid profile, the patient was diagnosed with Guillain-Barré syndrome, with concern for an acute motor axonal neuropathy variant. He was evaluated by neurology and the medical ICU teams. In accordance with standard ICU admission and triage principles, his preserved respiratory function, stable hemodynamics, absence of bulbar symptoms, and reassuring pulmonary mechanics supported admission to a step-down unit rather than ICU‑level care [1]. He was treated with intravenous immunoglobulin (0.4 g/kg/day for five days) and underwent serial monitoring of negative inspiratory force and vital capacity. Supportive care included physical and occupational therapy, deep vein thrombosis prophylaxis with subcutaneous heparin, management of hypertension, and correction of hyperkalemia with sodium zirconium cyclosilicate. Throughout hospitalization, he remained hemodynamically stable and did not develop respiratory failure or autonomic instability. Weakness stabilized with mild improvement in proximal strength, although significant distal lower‑extremity weakness persisted. After completion of immunotherapy and clinical stabilization, he was transferred to acute inpatient rehabilitation for continued motor recovery. 

## Discussion

GBS is an acute autoimmune neuropathy characterized by immune‑mediated injury to the peripheral nerves, leading to rapidly progressive symptoms such as paresthesias, muscle weakness, and, in severe cases, paralysis requiring close respiratory monitoring or intensive care support [1]. Although its precise cause remains unclear, the disorder is widely understood to result from aberrant immune activation following antecedent respiratory or gastrointestinal infection, with molecular mimicry between microbial antigens and peripheral nerve components believed to play a central role [1-4].

HCoV‑229E is an endemic alphacoronavirus that typically produces self‑limited upper respiratory tract symptoms. Despite its high prevalence - accounting for up to 15-20% of common cold presentations - neurologic complications are, to our knowledge, rarely reported. While GBS has been associated with several viral infections, including Epstein-Barr virus, HIV, and SARS‑CoV‑2, its occurrence following HCoV‑229E infection is uncommon, with only limited reports suggesting such a link [5-7].

First isolated in 1966, HCoV‑229E is thought to originate from African hipposiderid bats, with camelids serving as intermediate hosts. Its evolutionary history shares features with that of Middle East respiratory syndrome coronavirus (MERS-CoV). Although infection typically causes mild upper respiratory symptoms in healthy adults, young children, older adults, and immunocompromised individuals may develop severe lower respiratory tract disease. The virus has also been isolated in neural cells, raising the possibility of neurotropism; a preceding infectious trigger remains clinically meaningful. Establishing a temporal association supports the post‑infectious immune hypothesis and helps distinguish GBS from other immune‑mediated neuropathies, such as multiple sclerosis and chronic inflammatory demyelinating polyneuropathy (CIDP), which follow different clinical courses and may require alternative management strategies [5,6].

This case also emphasizes that diagnosis alone should not determine level of care. Although up to 30% of patients with GBS require mechanical ventilation, respiratory decline is best predicted by objective physiologic parameters rather than diagnostic category [1]. Despite marked distal weakness and electrophysiologic findings concerning an axonal variant, the patient maintained stable pulmonary mechanics, with preserved negative inspiratory force and vital capacity throughout hospitalization. Admission to an intermediate‑care setting allowed appropriate monitoring without unnecessary escalation to intensive care, supporting efficient use of critical care resources while maintaining patient safety.

Taken together, this case demonstrated two clinically relevant themes: the potential for seasonal coronaviruses such as HCoV‑229E to act as immunologic triggers for GBS, and the importance of physiologically guided triage in the management of acute neuromuscular weakness [8,9]. Further investigation is needed to clarify the incidence and mechanisms of coronavirus‑associated peripheral neuropathies beyond SARS‑CoV‑2.

## Conclusions

We report a case of Guillain-Barré syndrome following human coronavirus 229E infection that was successfully managed with intravenous immunoglobulin and careful physiologic monitoring without the need for intensive care admission. This case underscores the importance of early recognition, diagnostic confirmation, vigilant respiratory assessment, and evidence-based triage in patients with suspected GBS. It further highlights the value of evidence-based, physiology-driven triage in guiding level-of-care decisions while maintaining patient safety.
